# Crystal structure of dilead(II) oxochromate(VI) oxotellurate(IV)

**DOI:** 10.1107/S2056989017006995

**Published:** 2017-05-16

**Authors:** Matthias Weil

**Affiliations:** a Institute for Chemical Technologies and Analytics, Division of Structural Chemistry, Vienna University of Technology, Getreidemarkt 9/164-SC, A-1060 Vienna, Austria

**Keywords:** crystal structure, lead, oxochromate(VI), oxotellurate(IV), isotypism

## Abstract

Reaction of chromium(III) precursors with TeO_2_ in PbF_2_/PbO melts in air led to oxidation of chromium(III) to chromium(VI), whereas tellurium remained its oxidation state of IV. In the resulting title compound, Pb_2_(CrO_4_)(TeO_3_), the two types of anions are isolated from each other, hence a double salt is formed. The two independent Pb^2+^ cations exhibit coordination number nine under formation of very distorted coordination polyhedra [bond-length range = 2.363 (6)–3.276 (7) Å]. The oxochromate(VI) and oxotellurate(IV) anions have tetra­hedral and trigonal–pyramidal configurations, respectively. In the crystal structure, (001) layers of metal cations alternate with layers of TeO_3_^2−^ and CrO_4_^2−^ anions along [001], forming a three-dimensional framework structure. Pb_2_(CrO_4_)(TeO_3_) is isotypic with its sulfate analogue Pb_2_(SO_4_)(TeO_3_) and is comparatively discussed.

## Abstract 2

The crystal structure of Pb_2_(CrO_4_)(TeO_3_) is isotypic with its sulfate analogue Pb_2_(SO_4_)(TeO_3_). Comparison between the structures is made with the *COMPSTRU* program.

## Chemical context   

Pb_3_Fe_2_Te_2_O_12_ is an oxotellurate(VI) with inter­esting structural features. It crystallizes in the non-centrosymmetric space group *Cc* and has Te^VI^ and Fe^III^ atoms occupationally disordered at the same sites (Müller-Buschbaum & Wedel, 1997 ▸). This compound has been prepared by solid-state reactions from a PbO, Fe_2_O_3_ and TeO_2_ mixture in air, which led to oxidation of Te^IV^ to Te^VI^. During an attempt to replace iron(III) by chromium(III) to prepare a possible phase with composition ‘Pb_3_Cr_2_Te_2_O_12_’, the title compound, Pb_2_(CrO_4_)(TeO_3_), was obtained instead while working under similar conditions. Inter­estingly, chromium was then oxidized (Cr^III^ → Cr^VI^) while tellurium remained its oxidation state of IV. Pb_2_(CrO_4_)(TeO_3_) is isotypic with its sulfate analogue Pb_2_(SO_4_)(TeO_3_) (Weil & Shirkhanlou, 2017 ▸).

## Structural commentary   

All atoms in the asymmetric unit, *viz*. two Pb, one Cr, one Te and seven O sites, are located on general positions.

**Figure 1:**
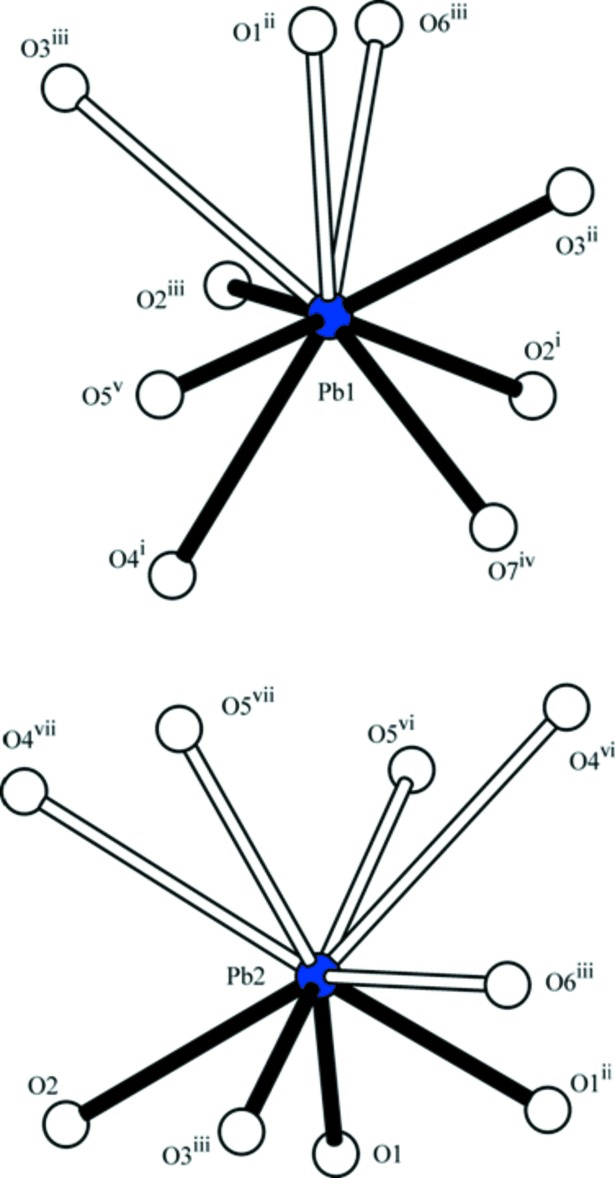
Coordination environments around the two Pb^2+^ cations in Pb_2_(CrO_4_)(TeO_3_). Pb—O bonds < 2.8 Å are given in full and longer Pb–O bonds are open. Symmetry operators refer to Table 1 ▸.

The coordination environments of the two Pb^2+^ cations are markedly different. If only Pb—O bond lengths < 2.8 Å are considered, atom Pb1 is surrounded by six O atoms in the range 2.4–2.8 Å whereas atom Pb2 has four oxygen atoms as coordination partners, three at ∼2.38 Å and one at 2.75 Å. Taking into account the more remote oxygen atoms as well, the coordination numbers are increased to nine for both Pb^2+^ cations (Fig. 1 ▸, Table 1 ▸).

The chromium atom shows a tetra­hedral and the tellurium a trigonal–pyramidal coordination by oxygen atoms. These two coordination polyhedra and the corresponding bond lengths ranges are typical for oxochromates(VI) (Pressprich *et al.*, 1988 ▸) and oxotellurates(IV) (Christy *et al.*, 2016 ▸), respectively.

**Figure 2:**
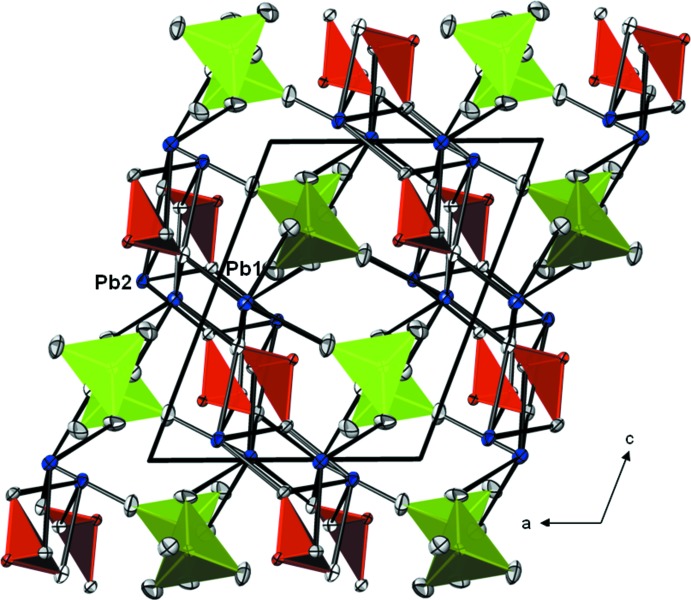
The crystal structure of Pb_2_(CrO_4_)(TeO_3_) in a projection along [010]. Te atoms and TeO_3_^2–^ trigonal pyramids are given in red, CrO_4_^2−^ tetra­hedra in green, Pb^2+^ cations in blue, O atoms are colourless. For clarity, only Pb—O bonds < 2.8 Å are displayed. Displacement ellipsoids are given at the 50% probability level.

In the crystal structure, the Pb^2+^ cations are arranged in layers parallel to (001) at *z* ∼ 0, ½ and in turn are stacked into columns extending along [010]. The two types of anion polyhedra are isolated and are likewise arranged into columnar arrangements along [010], forming anion layers situated at *z* ∼ ¼ and ¾. The metal cation and anion layers alternate along [001] and build up the three-dimensional framework of the crystal structure. The 5*s* ^2^ and 6*s* ^2^ electron lone pairs of the Te^IV^ atoms of the oxotellurate anions and of the Pb^2+^ cations, respectively, are stereochemically active and point into channels running parallel to the two types of columns along [010] (Fig. 2 ▸).

Relevant bond lengths of isotypic Pb_2_(CrO_4_)(TeO_3_) and Pb_2_(SO_4_)(TeO_3_) are compared in Table 1 ▸. Whereas the TeO_3_^2−^ anions in the two structures show only marginal differences, the expected differences in the *X*—O bond lengths (*X* = Cr, S) of the chromate and sulfate tetra­hedra (average values 1.65 and 1.48 Å, respectively) also have consequences for those Pb—O bonds where the corresponding atoms O4–O7 are involved. These Pb—O bonds differ by up to 0.20 Å. A more qu­anti­tative comparison of the two isotypic structures was made with the program *COMPSTRU (*de la Flor *et al.*, 2016 ▸). The degree of lattice distortion, *S*, is the spontaneous strain (sum of the squared eigenvalues of the strain tensor divided by 3) and amounts to 0.007. The maximum distance shows the maximal displacement between atomic positions of paired atoms and is 0.31 Å for atom pair O4. The next largest distances are 0.23 Å for pair O6, 0.17 Å for O5 and 0.13 Å for O7. The pairs of heavy atoms and the Cr/S pair show comparatively small distances of 0.095 Å (Pb1), 0.061 Å (Pb2), 0.087 Å (Te1) and 0.095 Å (Cr1/S1). The arithmetic mean of the distances is 0.12 Å. The measure of similarity (Δ) (Bergerhoff *et al.*, 1999 ▸) is 0.034, revealing a close relation between the two structures. Δ takes into consideration the differences in atomic positions and the ratios of the corresponding lattice parameters of the structures.

## Synthesis and crystallization   

Cr(NO_3_)_3_·9H_2_O, PbF_2_, PbO and TeO_2_ were mixed thoroughly in a stoichiometric ratio of 1:1:2:1 and heated in an open alumina crucible to 1033 K within six h, held at this temperature for 30 h and cooled within eight h to room temperature. Most of the material had evaporated, and only a few orange plates of the title compound were left.

Alternatively, replacement of Cr(NO_3_)_3_·9H_2_O with Cr_2_O_3_ under the same reaction conditions likewise led to the formation of Pb_2_(CrO_4_)(TeO_3_).

## Refinement   

Crystal data, data collection and structure refinement details are summarized in Table 2 ▸. Starting coordinates were taken from isotypic Pb_2_(SO_4_)(TeO_3_) (Weil & Shirkhanlou, 2017 ▸). The maximum and minimum electron densities are located 1.26 and 0.81 Å, respectively, from atom Pb2.

## Supplementary Material

Crystal structure: contains datablock(s) I, global. DOI: 10.1107/S2056989017006995/hb7677sup1.cif

Structure factors: contains datablock(s) I. DOI: 10.1107/S2056989017006995/hb7677Isup2.hkl

CCDC reference: 1548953

Additional supporting information:  crystallographic information; 3D view; checkCIF report

## Figures and Tables

**Table 1:** Comparison of bond lengths between isotypic Pb_2_(CrO_4_)(TeO_3_) and Pb_2_(SO_4_)(TeO_3_)

Bond	Pb_2_(CrO_4_)(TeO_3_)	Pb_2_(SO_4_)(TeO_3_)
Pb1—O2^i^	2.429 (6)	2.397 (3)
Pb1—O3^ii^	2.573 (6)	2.594 (3)
Pb1—O2^iii^	2.594 (6)	2.536 (3)
Pb1—O7^iv^	2.617 (7)	2.632 (3)
Pb1—O5^v^	2.750 (7)	2.789 (3)
Pb1—O4^i^	2.777 (7)	2.677 (3)
Pb1—O6^iii^	2.850 (7)	3.107 (4)
Pb1—O1^ii^	2.968 (6)	2.993 (3)
Pb1—O3^iii^	3.170 (6)	3.206 (3)
Pb2—O3^iii^	2.363 (6)	2.335 (3)
Pb2—O1^ii^	2.390 (6)	2.375 (3)
Pb2—O1	2.410 (6)	2.384 (3)
Pb2—O2	2.746 (6)	2.753 (3)
Pb2—O5^vi^	2.956 (7)	2.981 (4)
Pb2—O4^vii^	3.128 (7)	3.029 (3)
Pb2—O6^iii^	3.176 (7)	3.164 (3)
Pb2—O5^vii^	3.225 (7)	3.200 (4)
Pb2—O4^vi^	3.276 (7)	3.455 (3)
Te1—O2	1.891 (6)	1.890 (2)
Te1—O3	1.901 (6)	1.878 (2)
Te1—O1	1.902 (6)	1.895 (3)
Cr1—O7	1.634 (7)	1.462 (3)
Cr1—O5	1.640 (7)	1.476 (3)
Cr1—O4	1.653 (7)	1.488 (3)
Cr1—O6	1.667 (7)	1.484 (3)

Symmetry codes: (i) *x* − 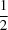, −*y* + 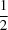, *z* − 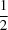; (ii) −*x* + 2, −*y* + 1, −*z* + 1; (iii) −*x* + 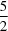, *y* − 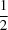, −*z* + 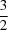; (iv) −*x* + 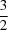, *y* − 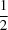, −*z* + 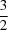; (v) −*x* + 2, −*y* + 1, −*z* + 2; (vi) *x*, *y*, *z* − 1; (vii) −*x* + 3, −*y* + 1, −*z* + 2.

**Table 2:** Experimental details

Crystal data	
Chemical formula	Pb_2_(CrO_4_)(TeO_3_)
*M*_r_	705.98
Crystal system, space group	Monoclinic, *P*2_1_/*n*
Temperature (K)	296
*a*, *b*, *c* (Å)	7.4736 (12), 10.8091 (16), 9.4065 (14)
β (°)	111.098 (12)
*V* (Å^3^)	708.95 (19)
*Z*	4
Radiation type	Mo *K*α
μ (mm^−1^)	52.91
Crystal size (mm)	0.09 × 0.06 × 0.01

Data collection	
Diffractometer	Bruker APEXII CCD
Absorption correction	Multi-scan (*SADABS*; Bruker, 2015 ▸)
*T*_min_, *T*_max_	0.264, 0.494
No. of measured, independent and observed [*I* > 2σ(*I*)] reflections	23485, 2183, 1760
*R*_int_	0.094
(sin θ/λ)_max_ (Å^−1^)	0.717

Refinement	
*R*[*F* ^2^ > 2σ(*F* ^2^)], *wR*(*F* ^2^), *S*	0.031, 0.070, 1.06
No. of reflections	2183
No. of parameters	100
Δρ_max_, Δρ_min_ (e Å^−3^)	2.47, −2.33

Computer programs: *APEX3* and *SAINT* (Bruker, 2015 ▸), *SHELXL2014* (Sheldrick, 2015 ▸), *ATOMS* (Dowty, 2006 ▸) and *publCIF* (Westrip, 2010 ▸).

## supplementary crystallographic information

### Crystal data

**Table:**

Pb_2_(CrO_4_)(TeO_3_)	*F*(000) = 1184
*M**_r_* = 705.98	*D*_x_ = 6.614 Mg m^−^^3^
Monoclinic, *P*2_1_/*n*	Mo *K*α radiation, λ = 0.71073 Å
*a* = 7.4736 (12) Å	Cell parameters from 3031 reflections
*b* = 10.8091 (16) Å	θ = 3.1–28.3°
*c* = 9.4065 (14) Å	µ = 52.91 mm^−^^1^
β = 111.098 (12)°	*T* = 296 K
*V* = 708.95 (19) Å^3^	Plate, orange
*Z* = 4	0.09 × 0.06 × 0.01 mm

### Data collection

**Table:**

Bruker APEXII CCD diffractometer	1760 reflections with *I* > 2σ(*I*)
ω– and φ–scans	*R*_int_ = 0.094
Absorption correction: multi-scan (*SADABS*; Bruker, 2015)	θ_max_ = 30.7°, θ_min_ = 3.0°
*T*_min_ = 0.264, *T*_max_ = 0.494	*h* = −10→10
23485 measured reflections	*k* = −15→15
2183 independent reflections	*l* = −13→13

### Refinement

**Table:**

Refinement on *F*^2^	100 parameters
Least-squares matrix: full	0 restraints
*R*[*F*^2^ > 2σ(*F*^2^)] = 0.031	*w* = 1/[σ^2^(*F*_o_^2^) + (0.0264*P*)^2^] where *P* = (*F*_o_^2^ + 2*F*_c_^2^)/3
*wR*(*F*^2^) = 0.070	(Δ/σ)_max_ = 0.001
*S* = 1.06	Δρ_max_ = 2.47 e Å^−^^3^
2183 reflections	Δρ_min_ = −2.33 e Å^−^^3^

### Special details

**Table:**

Geometry. All esds (except the esd in the dihedral angle between two l.s. planes) are estimated using the full covariance matrix. The cell esds are taken into account individually in the estimation of esds in distances, angles and torsion angles; correlations between esds in cell parameters are only used when they are defined by crystal symmetry. An approximate (isotropic) treatment of cell esds is used for estimating esds involving l.s. planes.

### Fractional atomic coordinates and isotropic or equivalent isotropic displacement parameters (Å^2^)

**Table:**

	*x*	*y*	*z*	*U*_iso_*/*U*_eq_	
Pb1	0.86662 (5)	0.16252 (3)	0.49204 (4)	0.01746 (9)	
Pb2	1.27667 (5)	0.45615 (3)	0.56450 (4)	0.01735 (9)	
Te1	1.11378 (8)	0.63302 (5)	0.81301 (6)	0.01180 (12)	
Cr1	1.2298 (2)	0.61161 (13)	1.21196 (16)	0.0152 (3)	
O1	1.0417 (9)	0.5911 (6)	0.6035 (7)	0.0187 (13)	
O2	1.3339 (8)	0.5323 (5)	0.8562 (7)	0.0159 (12)	
O3	1.2413 (9)	0.7803 (5)	0.7920 (6)	0.0153 (12)	
O4	1.3081 (10)	0.4675 (6)	1.2262 (8)	0.0295 (17)	
O5	1.3146 (11)	0.6714 (6)	1.3836 (8)	0.0292 (16)	
O6	1.3082 (11)	0.6927 (7)	1.0953 (8)	0.0331 (18)	
O7	0.9957 (10)	0.6054 (7)	1.1419 (9)	0.0337 (18)	

### Atomic displacement parameters (Å^2^)

**Table:**

	*U*^11^	*U*^22^	*U*^33^	*U*^12^	*U*^13^	*U*^23^
Pb1	0.01755 (19)	0.01525 (15)	0.01860 (18)	0.00102 (12)	0.00530 (14)	−0.00226 (12)
Pb2	0.01405 (18)	0.01770 (16)	0.01959 (18)	0.00177 (12)	0.00520 (14)	0.00440 (12)
Te1	0.0107 (3)	0.0117 (2)	0.0124 (3)	0.00001 (19)	0.0036 (2)	−0.00064 (19)
Cr1	0.0131 (7)	0.0191 (7)	0.0138 (7)	−0.0005 (5)	0.0053 (6)	−0.0002 (5)
O1	0.013 (3)	0.025 (3)	0.015 (3)	0.003 (3)	0.002 (3)	−0.004 (3)
O2	0.014 (3)	0.009 (3)	0.024 (3)	0.003 (2)	0.005 (3)	0.002 (2)
O3	0.018 (3)	0.013 (3)	0.014 (3)	−0.003 (2)	0.005 (3)	0.000 (2)
O4	0.032 (4)	0.024 (3)	0.031 (4)	0.003 (3)	0.010 (3)	−0.006 (3)
O5	0.041 (4)	0.028 (4)	0.018 (3)	0.007 (3)	0.010 (3)	−0.008 (3)
O6	0.040 (5)	0.036 (4)	0.029 (4)	−0.018 (3)	0.019 (4)	0.004 (3)
O7	0.017 (4)	0.039 (4)	0.043 (5)	0.005 (3)	0.010 (3)	0.015 (4)

### Geometric parameters (Å, º)

**Table:**

Pb1—O2^i^	2.429 (6)	Cr1—Te1^x^	3.8465 (15)
Pb1—O3^ii^	2.573 (6)	Cr1—Pb1^xi^	3.9532 (15)
Pb1—O2^iii^	2.594 (6)	Cr1—Pb1^v^	3.9593 (14)
Pb1—O7^iv^	2.617 (7)	Cr1—Pb1^xii^	4.1534 (15)
Pb1—O5^v^	2.750 (7)	O1—Pb2^ii^	2.390 (6)
Pb1—O4^i^	2.777 (7)	O1—Pb1^ii^	2.968 (6)
Pb1—O6^iii^	2.850 (7)	O1—Pb1^xi^	4.515 (6)
Pb1—O1^ii^	2.968 (6)	O2—Pb1^xi^	2.429 (6)
Pb1—O3^iii^	3.170 (6)	O2—Pb1^xii^	2.594 (6)
Pb1—Te1^iii^	3.6630 (9)	O2—Pb1^ii^	4.508 (6)
Pb2—O3^iii^	2.363 (6)	O3—Pb2^xii^	2.363 (6)
Pb2—O1^ii^	2.390 (6)	O3—Pb1^ii^	2.573 (6)
Pb2—O1	2.410 (6)	O3—Pb1^xii^	3.170 (6)
Pb2—O2	2.746 (6)	O4—Pb1^xi^	2.777 (7)
Pb2—O5^vi^	2.956 (7)	O4—Pb2^vii^	3.128 (7)
Pb2—O4^vii^	3.128 (7)	O4—Te1^v^	3.228 (7)
Pb2—O6^iii^	3.176 (7)	O4—Pb2^ix^	3.276 (7)
Pb2—O5^vii^	3.225 (7)	O4—Pb1^xii^	4.262 (7)
Pb2—O4^vi^	3.276 (7)	O5—Pb1^v^	2.750 (7)
Pb2—Te1	3.5567 (7)	O5—Pb2^ix^	2.956 (7)
Te1—O2	1.891 (6)	O5—Pb2^vii^	3.225 (7)
Te1—O3	1.901 (6)	O5—Te1^x^	3.314 (8)
Te1—O1	1.902 (6)	O6—Pb1^xii^	2.850 (7)
Te1—O6	2.608 (7)	O6—Te1^x^	3.097 (7)
Te1—O7^v^	2.782 (7)	O6—Pb2^xii^	3.176 (7)
Te1—O6^viii^	3.097 (7)	O6—Pb2^vii^	3.912 (8)
Cr1—O7	1.634 (7)	O6—Pb1^xi^	4.023 (8)
Cr1—O5	1.640 (7)	O7—Pb1^xiii^	2.617 (7)
Cr1—O4	1.653 (7)	O7—Te1^v^	2.782 (7)
Cr1—O6	1.667 (7)	O7—Pb2^v^	4.032 (8)
Cr1—Pb2^vii^	3.6027 (16)	O7—Pb1^v^	4.081 (8)
Cr1—Pb2^ix^	3.6204 (15)	O7—Pb2^ix^	4.099 (7)
Cr1—Te1^v^	3.6339 (16)	O7—Pb1^xi^	4.568 (8)
			
O2^i^—Pb1—O3^ii^	74.19 (19)	O7—Cr1—Pb1^xi^	101.5 (3)
O2^i^—Pb1—O2^iii^	73.9 (2)	O5—Cr1—Pb1^xi^	136.8 (3)
O3^ii^—Pb1—O2^iii^	126.0 (2)	O4—Cr1—Pb1^xi^	35.4 (3)
O2^i^—Pb1—O7^iv^	69.6 (2)	O6—Cr1—Pb1^xi^	80.3 (3)
O3^ii^—Pb1—O7^iv^	70.9 (2)	Te1—Cr1—Pb1^xi^	60.35 (2)
O2^iii^—Pb1—O7^iv^	132.5 (2)	Pb2^vii^—Cr1—Pb1^xi^	75.50 (3)
O2^i^—Pb1—O5^v^	144.9 (2)	Pb2^ix^—Cr1—Pb1^xi^	99.99 (3)
O3^ii^—Pb1—O5^v^	105.45 (19)	Te1^v^—Cr1—Pb1^xi^	74.38 (3)
O2^iii^—Pb1—O5^v^	125.4 (2)	Te1^x^—Cr1—Pb1^xi^	110.89 (4)
O7^iv^—Pb1—O5^v^	77.1 (2)	O7—Cr1—Pb1^v^	82.5 (3)
O2^i^—Pb1—O4^i^	87.9 (2)	O5—Cr1—Pb1^v^	33.7 (3)
O3^ii^—Pb1—O4^i^	149.93 (19)	O4—Cr1—Pb1^v^	132.3 (3)
O2^iii^—Pb1—O4^i^	68.9 (2)	O6—Cr1—Pb1^v^	109.6 (3)
O7^iv^—Pb1—O4^i^	80.3 (2)	Te1—Cr1—Pb1^v^	131.89 (4)
O5^v^—Pb1—O4^i^	75.6 (2)	Pb2^vii^—Cr1—Pb1^v^	97.02 (3)
O2^i^—Pb1—O6^iii^	83.2 (2)	Pb2^ix^—Cr1—Pb1^v^	67.93 (3)
O3^ii^—Pb1—O6^iii^	69.8 (2)	Te1^v^—Cr1—Pb1^v^	101.01 (4)
O2^iii^—Pb1—O6^iii^	64.2 (2)	Te1^x^—Cr1—Pb1^v^	72.07 (3)
O7^iv^—Pb1—O6^iii^	136.9 (2)	Pb1^xi^—Cr1—Pb1^v^	167.69 (4)
O5^v^—Pb1—O6^iii^	130.5 (2)	O7—Cr1—Pb1^xii^	132.0 (3)
O4^i^—Pb1—O6^iii^	132.9 (2)	O5—Cr1—Pb1^xii^	108.4 (3)
O2^i^—Pb1—O1^ii^	127.50 (19)	O4—Cr1—Pb1^xii^	82.4 (3)
O3^ii^—Pb1—O1^ii^	59.35 (17)	O6—Cr1—Pb1^xii^	30.7 (3)
O2^iii^—Pb1—O1^ii^	113.96 (17)	Te1—Cr1—Pb1^xii^	56.17 (2)
O7^iv^—Pb1—O1^ii^	112.2 (2)	Pb2^vii^—Cr1—Pb1^xii^	62.31 (3)
O5^v^—Pb1—O1^ii^	75.3 (2)	Pb2^ix^—Cr1—Pb1^xii^	129.63 (4)
O4^i^—Pb1—O1^ii^	144.43 (19)	Te1^v^—Cr1—Pb1^xii^	132.87 (4)
O6^iii^—Pb1—O1^ii^	59.76 (19)	Te1^x^—Cr1—Pb1^xii^	54.39 (2)
O2^i^—Pb1—O3^iii^	125.43 (18)	Pb1^xi^—Cr1—Pb1^xii^	59.33 (2)
O3^ii^—Pb1—O3^iii^	116.15 (16)	Pb1^v^—Cr1—Pb1^xii^	126.12 (4)
O2^iii^—Pb1—O3^iii^	56.62 (16)	Te1—O1—Pb2^ii^	125.4 (3)
O7^iv^—Pb1—O3^iii^	164.00 (19)	Te1—O1—Pb2	110.6 (3)
O5^v^—Pb1—O3^iii^	87.05 (19)	Pb2^ii^—O1—Pb2	112.1 (2)
O4^i^—Pb1—O3^iii^	93.90 (18)	Te1—O1—Pb1^ii^	95.2 (2)
O6^iii^—Pb1—O3^iii^	56.72 (18)	Pb2^ii^—O1—Pb1^ii^	106.0 (2)
O1^ii^—Pb1—O3^iii^	64.71 (16)	Pb2—O1—Pb1^ii^	103.7 (2)
O2^i^—Pb1—Te1^iii^	94.34 (14)	Te1—O1—Pb1^xi^	55.69 (15)
O3^ii^—Pb1—Te1^iii^	114.85 (13)	Pb2^ii^—O1—Pb1^xi^	121.5 (2)
O2^iii^—Pb1—Te1^iii^	29.32 (12)	Pb2—O1—Pb1^xi^	63.04 (13)
O7^iv^—Pb1—Te1^iii^	161.34 (16)	Pb1^ii^—O1—Pb1^xi^	132.40 (18)
O5^v^—Pb1—Te1^iii^	116.07 (15)	Te1—O2—Pb1^xi^	121.8 (3)
O4^i^—Pb1—Te1^iii^	90.00 (15)	Te1—O2—Pb1^xii^	108.5 (2)
O6^iii^—Pb1—Te1^iii^	45.10 (15)	Pb1^xi^—O2—Pb1^xii^	106.1 (2)
O1^ii^—Pb1—Te1^iii^	84.89 (12)	Te1—O2—Pb2	98.5 (2)
O3^iii^—Pb1—Te1^iii^	31.26 (10)	Pb1^xi^—O2—Pb2	102.47 (19)
O3^iii^—Pb2—O1^ii^	87.6 (2)	Pb1^xii^—O2—Pb2	120.4 (2)
O3^iii^—Pb2—O1	101.9 (2)	Te1—O2—Pb1^ii^	52.16 (15)
O1^ii^—Pb2—O1	67.9 (2)	Pb1^xi^—O2—Pb1^ii^	163.9 (2)
O3^iii^—Pb2—O2	72.00 (18)	Pb1^xii^—O2—Pb1^ii^	89.88 (14)
O1^ii^—Pb2—O2	119.0 (2)	Pb2—O2—Pb1^ii^	66.54 (12)
O1—Pb2—O2	61.76 (19)	Te1—O3—Pb2^xii^	118.7 (3)
O3^iii^—Pb2—O5^vi^	177.4 (2)	Te1—O3—Pb1^ii^	109.1 (2)
O1^ii^—Pb2—O5^vi^	93.8 (2)	Pb2^xii^—O3—Pb1^ii^	109.8 (2)
O1—Pb2—O5^vi^	80.6 (2)	Te1—O3—Pb1^xii^	88.8 (2)
O2—Pb2—O5^vi^	109.06 (17)	Pb2^xii^—O3—Pb1^xii^	100.75 (19)
O3^iii^—Pb2—O4^vii^	95.8 (2)	Pb1^ii^—O3—Pb1^xii^	129.3 (2)
O1^ii^—Pb2—O4^vii^	176.63 (19)	Te1—O3—Pb2	58.39 (14)
O1—Pb2—O4^vii^	110.89 (19)	Pb2^xii^—O3—Pb2	176.2 (2)
O2—Pb2—O4^vii^	61.95 (18)	Pb1^ii^—O3—Pb2	73.89 (13)
O5^vi^—Pb2—O4^vii^	82.9 (2)	Pb1^xii^—O3—Pb2	77.08 (11)
O3^iii^—Pb2—O6^iii^	60.41 (19)	Cr1—O4—Pb1^xi^	124.4 (4)
O1^ii^—Pb2—O6^iii^	60.90 (19)	Cr1—O4—Pb2^vii^	92.6 (3)
O1—Pb2—O6^iii^	125.6 (2)	Pb1^xi^—O4—Pb2^vii^	103.2 (2)
O2—Pb2—O6^iii^	132.40 (18)	Cr1—O4—Te1^v^	90.3 (3)
O5^vi^—Pb2—O6^iii^	118.53 (18)	Pb1^xi^—O4—Te1^v^	99.5 (2)
O4^vii^—Pb2—O6^iii^	121.22 (17)	Pb2^vii^—O4—Te1^v^	150.2 (3)
O3^iii^—Pb2—O5^vii^	79.3 (2)	Cr1—O4—Pb2^ix^	88.1 (3)
O1^ii^—Pb2—O5^vii^	132.23 (19)	Pb1^xi^—O4—Pb2^ix^	147.0 (2)
O1—Pb2—O5^vii^	159.74 (19)	Pb2^vii^—O4—Pb2^ix^	78.49 (16)
O2—Pb2—O5^vii^	100.28 (16)	Te1^v^—O4—Pb2^ix^	71.93 (15)
O5^vi^—Pb2—O5^vii^	98.15 (17)	Cr1—O4—Te1	60.7 (2)
O4^vii^—Pb2—O5^vii^	49.20 (17)	Pb1^xi^—O4—Te1	64.28 (14)
O6^iii^—Pb2—O5^vii^	72.86 (18)	Pb2^vii^—O4—Te1	114.8 (2)
O3^iii^—Pb2—O4^vi^	128.57 (18)	Te1^v^—O4—Te1	92.34 (16)
O1^ii^—Pb2—O4^vi^	76.61 (19)	Pb2^ix^—O4—Te1	145.4 (2)
O1—Pb2—O4^vi^	115.84 (19)	Cr1—O4—Pb1^xii^	75.0 (3)
O2—Pb2—O4^vi^	156.81 (17)	Pb1^xi^—O4—Pb1^xii^	65.68 (15)
O5^vi^—Pb2—O4^vi^	49.92 (17)	Pb2^vii^—O4—Pb1^xii^	64.16 (13)
O4^vii^—Pb2—O4^vi^	101.51 (16)	Te1^v^—O4—Pb1^xii^	144.5 (2)
O6^iii^—Pb2—O4^vi^	69.25 (17)	Pb2^ix^—O4—Pb1^xii^	137.7 (2)
O5^vii^—Pb2—O4^vi^	76.56 (18)	Te1—O4—Pb1^xii^	52.26 (9)
O3^iii^—Pb2—Te1	87.36 (14)	Cr1—O5—Pb1^v^	126.9 (4)
O1^ii^—Pb2—Te1	93.13 (15)	Cr1—O5—Pb2^ix^	100.0 (3)
O1—Pb2—Te1	30.03 (14)	Pb1^v^—O5—Pb2^ix^	96.0 (2)
O2—Pb2—Te1	31.73 (12)	Cr1—O5—Pb2^vii^	89.4 (3)
O5^vi^—Pb2—Te1	94.68 (13)	Pb1^v^—O5—Pb2^vii^	143.1 (2)
O4^vii^—Pb2—Te1	86.63 (13)	Pb2^ix^—O5—Pb2^vii^	81.85 (17)
O6^iii^—Pb2—Te1	137.38 (13)	Cr1—O5—Te1^x^	95.9 (3)
O5^vii^—Pb2—Te1	131.31 (12)	Pb1^v^—O5—Te1^x^	98.0 (2)
O4^vi^—Pb2—Te1	141.21 (12)	Pb2^ix^—O5—Te1^x^	146.0 (3)
O2—Te1—O3	94.3 (3)	Pb2^vii^—O5—Te1^x^	68.42 (15)
O2—Te1—O1	89.2 (3)	Cr1—O6—Te1	109.9 (3)
O3—Te1—O1	93.3 (3)	Cr1—O6—Pb1^xii^	131.9 (4)
O2—Te1—O6	78.5 (3)	Te1—O6—Pb1^xii^	84.2 (2)
O3—Te1—O6	77.4 (2)	Cr1—O6—Te1^x^	103.6 (3)
O1—Te1—O6	163.8 (2)	Te1—O6—Te1^x^	146.3 (3)
O2—Te1—O7^v^	73.4 (2)	Pb1^xii^—O6—Te1^x^	75.99 (17)
O3—Te1—O7^v^	167.7 (2)	Cr1—O6—Pb2^xii^	136.5 (4)
O1—Te1—O7^v^	87.0 (2)	Te1—O6—Pb2^xii^	78.27 (18)
O6—Te1—O7^v^	99.2 (2)	Pb1^xii^—O6—Pb2^xii^	90.68 (17)
O2—Te1—O6^viii^	150.4 (2)	Te1^x^—O6—Pb2^xii^	75.03 (16)
O3—Te1—O6^viii^	72.5 (2)	Cr1—O6—Pb2^vii^	67.0 (2)
O1—Te1—O6^viii^	66.0 (2)	Te1—O6—Pb2^vii^	135.9 (3)
O6—Te1—O6^viii^	122.06 (13)	Pb1^xii^—O6—Pb2^vii^	71.42 (16)
O7^v^—Te1—O6^viii^	118.5 (2)	Te1^x^—O6—Pb2^vii^	61.99 (13)
O7—Cr1—O5	113.0 (4)	Pb2^xii^—O6—Pb2^vii^	136.1 (2)
O7—Cr1—O4	106.9 (4)	Cr1—O6—Pb1^xi^	75.6 (3)
O5—Cr1—O4	106.9 (3)	Te1—O6—Pb1^xi^	65.61 (16)
O7—Cr1—O6	109.6 (4)	Pb1^xii^—O6—Pb1^xi^	69.10 (16)
O5—Cr1—O6	109.8 (4)	Te1^x^—O6—Pb1^xi^	128.5 (2)
O4—Cr1—O6	110.5 (4)	Pb2^xii^—O6—Pb1^xi^	139.7 (2)
O7—Cr1—Te1	76.0 (3)	Pb2^vii^—O6—Pb1^xi^	71.46 (13)
O5—Cr1—Te1	151.4 (3)	Cr1—O7—Pb1^xiii^	163.1 (4)
O4—Cr1—Te1	95.3 (3)	Cr1—O7—Te1^v^	107.9 (3)
O6—Cr1—Te1	43.8 (3)	Pb1^xiii^—O7—Te1^v^	89.0 (2)
O7—Cr1—Pb2^vii^	161.7 (3)	Cr1—O7—Te1	77.3 (3)
O5—Cr1—Pb2^vii^	63.5 (3)	Pb1^xiii^—O7—Te1	95.7 (2)
O4—Cr1—Pb2^vii^	60.2 (3)	Te1^v^—O7—Te1	113.1 (2)
O6—Cr1—Pb2^vii^	87.8 (3)	Cr1—O7—Pb2^v^	117.5 (3)
Te1—Cr1—Pb2^vii^	116.13 (4)	Pb1^xiii^—O7—Pb2^v^	71.26 (16)
O7—Cr1—Pb2^ix^	95.0 (3)	Te1^v^—O7—Pb2^v^	59.62 (13)
O5—Cr1—Pb2^ix^	53.5 (3)	Te1—O7—Pb2^v^	164.5 (2)
O4—Cr1—Pb2^ix^	64.8 (3)	Cr1—O7—Pb1^v^	74.1 (3)
O6—Cr1—Pb2^ix^	154.9 (3)	Pb1^xiii^—O7—Pb1^v^	99.4 (2)
Te1—Cr1—Pb2^ix^	155.08 (4)	Te1^v^—O7—Pb1^v^	116.1 (2)
Pb2^vii^—Cr1—Pb2^ix^	68.28 (3)	Te1—O7—Pb1^v^	128.5 (2)
O7—Cr1—Te1^v^	46.7 (3)	Pb2^v^—O7—Pb1^v^	63.88 (12)
O5—Cr1—Te1^v^	111.3 (3)	Cr1—O7—Pb2^ix^	61.6 (2)
O4—Cr1—Te1^v^	62.7 (3)	Pb1^xiii^—O7—Pb2^ix^	129.8 (2)
O6—Cr1—Te1^v^	138.4 (3)	Te1^v^—O7—Pb2^ix^	64.15 (14)
Te1—Cr1—Te1^v^	94.66 (4)	Te1—O7—Pb2^ix^	133.03 (19)
Pb2^vii^—Cr1—Te1^v^	116.17 (4)	Pb2^v^—O7—Pb2^ix^	58.65 (10)
Pb2^ix^—Cr1—Te1^v^	63.55 (3)	Pb1^v^—O7—Pb2^ix^	62.50 (11)
O7—Cr1—Te1^x^	136.4 (3)	Cr1—O7—Pb1^xi^	58.0 (2)
O5—Cr1—Te1^x^	59.0 (3)	Pb1^xiii^—O7—Pb1^xi^	129.7 (2)
O4—Cr1—Te1^x^	116.4 (3)	Te1^v^—O7—Pb1^xi^	72.81 (16)
O6—Cr1—Te1^x^	51.5 (3)	Te1—O7—Pb1^xi^	53.91 (10)
Te1—Cr1—Te1^x^	95.23 (3)	Pb2^v^—O7—Pb1^xi^	128.24 (17)
Pb2^vii^—Cr1—Te1^x^	59.04 (2)	Pb1^v^—O7—Pb1^xi^	130.79 (17)
Pb2^ix^—Cr1—Te1^x^	106.81 (4)	Pb2^ix^—O7—Pb1^xi^	83.91 (12)
Te1^v^—Cr1—Te1^x^	170.10 (4)		

Symmetry codes: (i) *x*−1/2, −*y*+1/2, *z*−1/2; (ii) −*x*+2, −*y*+1, −*z*+1; (iii) −*x*+5/2, *y*−1/2, −*z*+3/2; (iv) −*x*+3/2, *y*−1/2, −*z*+3/2; (v) −*x*+2, −*y*+1, −*z*+2; (vi) *x*, *y*, *z*−1; (vii) −*x*+3, −*y*+1, −*z*+2; (viii) *x*−1/2, −*y*+3/2, *z*−1/2; (ix) *x*, *y*, *z*+1; (x) *x*+1/2, −*y*+3/2, *z*+1/2; (xi) *x*+1/2, −*y*+1/2, *z*+1/2; (xii) −*x*+5/2, *y*+1/2, −*z*+3/2; (xiii) −*x*+3/2, *y*+1/2, −*z*+3/2.
